# Postnatal ketone deficiency impairs adult oocyte quality *via* enhancing histone acetylation in malnourished mice

**DOI:** 10.1016/j.jbc.2026.113535

**Published:** 2026-09-08

**Authors:** Xin-Ge Zhang, Ming-Zhi Li, Han Sha, Shi-Yao Tao, Yuan Qiao, Qi-Wen Yang, Li-Rong Wang, Xiao-Qian Zhang, Dan-Yang Chong, Zhi-Bin Hu, Yuan Lin, Xiu-Feng Ling, Chao-Jun Li

**Affiliations:** 1 State Key Laboratory of Reproductive Medicine and Offspring Health, Nanjing Medical University, Nanjing, China; 2 Women's Hospital of Nanjing Medical University, Nanjing Women and Children’s Healthcare Hospital, Nanjing, China; 3 Changzhou Maternity and Child Health Care Hospital, Changzhou Medical Center, Nanjing Medical University, Changzhou, China

**Keywords:** histone acetylation, oocyte, early-life, β-hydroxybutyrate, secondary nutritional stress

## Abstract

Early postnatal life is important for reproductive development, but the effects of early-life nutrition on female reproductive capacity remain largely unknown. Here, we investigated whether neonatal ketone body deficiency affects adult oocyte quality using a lactational malnutrition model and Hmgcs2-deficient mice. We found that impaired neonatal ketogenesis caused long-lasting impairments in female reproductive function, including reduced ovarian reserve, decreased oocyte developmental competence, altered hormone levels, and impaired embryo development. Neonatal β-hydroxybutyrate (β-HB) supplementation partially improved these reproductive abnormalities, indicating an important role of ketone metabolism during early ovarian development. Transcriptomic and epigenomic analyses showed that Hmgcs2 deficiency disrupted histone H3 acetylation homeostasis in oocytes. These Ace-H3 changes were enriched in genes related to oxidative stress and apoptosis and were accompanied by transcriptional alterations. β-HB supplementation partially restored the altered Ace-H3 patterns. Together, our findings reveal that ketone bodies regulate oocyte development by connecting early-life nutrition with epigenetic regulation.

Nutrition is integral to female reproductive health. Both undernutrition and overnutrition have been associated with reduced ovarian reserve and earlier menopause (1, 2, 3). Normal reproductive function depends on adequate nutritional support for the hypothalamic–pituitary–ovarian (HPO) axis, which sustains the synthesis and release of gonadotropin-releasing hormone (GnRH), luteinizing hormone (LH), follicle-stimulating hormone (FSH), and ovarian steroids (4, 5). Under conditions of limited energy availability, GnRH secretion is suppressed, sex steroid levels fall, follicular growth is impaired, and ovulation is disrupted (1). At the other extreme, overnutrition and obesity are linked to menstrual disorders, infertility, miscarriage, and adverse pregnancy outcomes, while chronic nutrient insufficiency likewise compromises fertility (6, 7).

Yet the timing of nutritional perturbation has not been adequately addressed. Much of the available evidence derives from exposures during adolescence or adulthood. The Developmental Origins of Health and Disease (DOHaD) framework, however, emphasizes that adverse conditions in early development—including infancy—can leave lasting imprints on organ development and later life disease risk (8, 9, 10). Early postnatal life features rapid metabolic reprogramming alongside widespread epigenetic resetting, creating a window in which nutritional state could be written into durable transcriptional programs (11, 12). Defining the metabolite-derived signals that translate transient deprivation into long-term cellular memory is therefore critical for understanding adult reproductive phenotypes. Whether the developing ovary uses such metabolic cues—and which ones—remains unclear.

Ketone body metabolism offers a tractable candidate mechanism. Ketone bodies, particularly β-hydroxybutyrate (β-HB), rise transiently after birth and serve not only as alternative fuels but also as signaling metabolites that can influence epigenetic regulation through protein lysine acetylation (K-ac) and β-hydroxybutyrylation (K-bhb), thereby affecting chromatin organization and gene transcription (13). We previously showed that Hmgcs2, which catalyzes a committed step in mitochondrial ketogenesis, is rapidly induced after birth by colostrum-derived fatty acids and declines thereafter (14, 15). This transient postnatal ketogenesis supports neonatal heart development by tuning mitochondrial protein acylation and promoting mitochondrial maturation (14). In the ovary, postnatal ketone deficiency is associated with excessive germ cell apoptosis and a reduced primordial follicle pool (15), raising the possibility that neonatal ketone availability contributes to long-term reproductive trajectories.

Here we test the hypothesis that early nutritional deprivation impairs adult oocyte quality and reproductive capacity by suppressing neonatal ketone production and altering epigenetic regulation during early oocyte development. Using a lactational undernutrition paradigm together with genetic disruption of ketogenesis, we examine how neonatal ketone deficiency affects ovarian development, oocyte competence, and reproductive resilience to subsequent nutritional stress.

## Results

### Nutrient restriction during lactation reduces ketone body metabolism and increases histone H3 acetylation in neonatal ovaries

We established a malnourished mouse model by increasing litter size to simulate insufficient milk intake from birth and investigate the effects of early-life malnutrition on ovarian function. Model validation showed that the body weight of malnourished female mice was significantly lower than that of control mice (Fig. 1*A*). Compared with the control group, malnourished mice showed reduced progesterone levels, whereas no significant differences were observed in follicle-stimulating hormone (FSH), luteinizing hormone (LH), or estradiol (E2) levels (Fig. 1, *B*–*E*).

**Figure 1:**
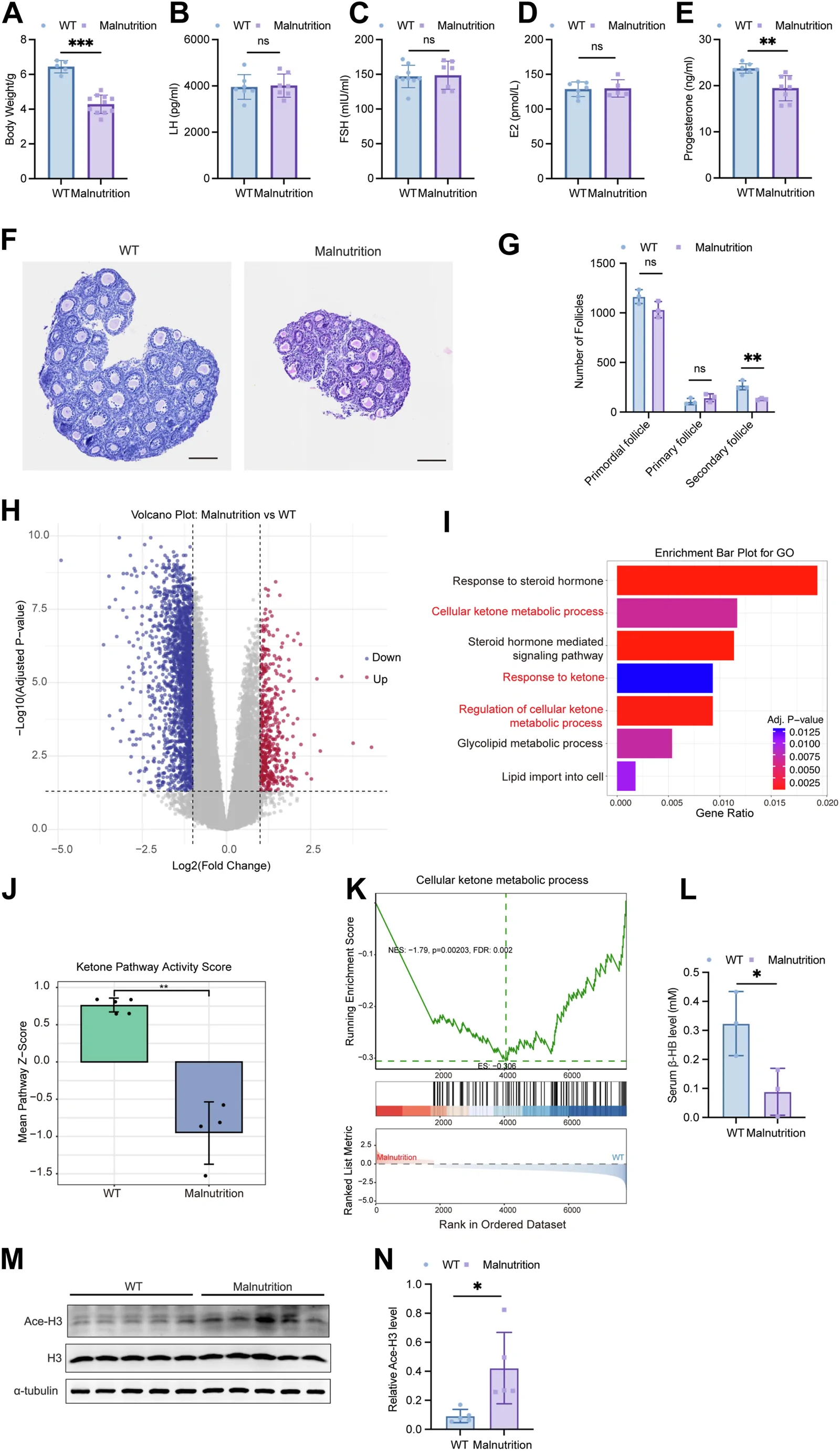
**Nutrient restriction during lactation alters ovarian ketone body metabolism in neonatal mice.***A*, body weight of female pups reared in WT and enlarged litters (malnutrition). *B–E*, serum progesterone, follicle-stimulating hormone (FSH), luteinizing hormone (LH) and estradiol (E2) levels in WT and malnutrition mice. *F*, representative images of H&E-stained ovarian sections from 14-day-old WT and Malnutrition group. Scale bar, 100 μm. *G*, quantification of the number of primordial, primary, and secondary follicles per ovary in WT and Malnutrition group. *H*, RNA sequencing of ovaries from postnatal day 7 mice showing differentially expressed genes (DEGs) between WT and malnutrition groups (|log_2_FC| > 0.75, adjusted *p* < 0.05). *I*, gene ontology enrichment analysis of DEGs. *J* and *K*, single-sample gene set enrichment analysis (ssGSEA) and gene set enrichment analysis (GSEA) of ketone body metabolism–related gene sets in ovaries. *L*, serum β-hydroxybutyrate (β-HB) levels in WT and malnutrition mice. *M*, Western blot analysis of Ace-H3 levels in P7 ovaries from control and malnourished mice. H3 was used as a loading control. *N*, quantification of Ace-H3 protein levels normalized to total H3. ∗*p* < 0.05. All data are presented as the mean ± S.D. ∗*p* < 0.05, ∗∗*p* < 0.01, ∗∗∗*p* < 0.001, ns, not significant, unpaired *t* test. *A*, WT (n = 5) malnutrition (n = 11). *B*, WT (n = 7) malnutrition (n = 7). *C*, WT (n = 9) malnutrition (n = 7). *D*, WT (n = 7) malnutrition (n = 5). *E*, WT (n = 7) malnutrition (n = 8). *F* and *G*, WT (n = 3) malnutrition (n = 3). *H-K*, WT (n = 5) malnutrition (n = 4). *L*, WT (n = 3) malnutrition (n = 3).

The impact of early-life malnutrition on ovarian morphology and folliculogenesis was assessed by hematoxylin and eosin (H&E) staining of ovarian sections from 14-day-old mice. Histological analysis revealed that ovaries from malnourished mice were noticeably smaller than those from control mice (Fig. 1*F*). Follicle counting further showed that the numbers of primordial and primary follicles were comparable between the two groups, whereas the number of secondary follicles was significantly reduced in the malnourished group (Fig. 1*G*), indicating impaired early follicle development.

We next collected ovaries from 7-day-old mice for RNA sequencing (RNA-seq) to explore the molecular mechanisms underlying impaired ovarian development. A total of 2523 differentially expressed genes (DEGs) were identified between the malnourished and control groups, using the criteria |log2FC| > 1 and adjusted *p* < 0.05. Among these DEGs, 457 genes were upregulated and 2066 genes were downregulated in malnourished ovaries (Fig. 1*H*). Gene Ontology (GO) enrichment analysis revealed that early-life malnutrition was primarily associated with response to steroid hormone, steroid hormone-mediated signaling pathway, cellular ketone metabolic process, response to ketone, and regulation of cellular ketone metabolic process (Fig. 1*I*). Single-sample gene set enrichment analysis (ssGSEA) and gene set enrichment analysis (GSEA) further revealed reduced ketone body metabolism in the ovaries of malnourished mice (Fig. 1, *J* and *K*).

Our previous studies showed a transient increase in ketone bodies in mice between postnatal days 1 and 7 (15). Consistently, serum β-HB measurement showed significantly lower blood ketone levels in malnourished mice than in control mice (Fig. 1*L*). Given the link between ketone body metabolism and histone acetylation regulation, we further examined whether nutrient restriction during lactation altered histone H3 acetylation in neonatal ovaries (16). Western blot analysis showed that Ace-H3 levels were significantly increased in P7 ovaries from malnourished mice compared with control mice (Fig. 1, *M* and *N*). These findings indicate that early-life malnutrition is accompanied by both reduced ketone body metabolism and increased ovarian histone H3 acetylation during the neonatal period. This observation also provides a basis for subsequent experiments using Hmgcs2-deficient mice to investigate whether impaired ketone body metabolism contributes to abnormal histone acetylation and oocyte defects.

In combination with the decreased progesterone levels observed in malnourished neonatal mice, these results suggest that early-life malnutrition disrupts the ovarian metabolic environment, particularly ketone body metabolism, and is associated with abnormal histone H3 acetylation, thereby potentially affecting early ovarian development.

### Ketone body supplementation restores malnutrition-induced impairment of early ovarian development

To assess the role of ketone bodies in early ovarian development, we injected β-HB intraperitoneally into malnourished and WT neonatal mice, named the malnutrition+β-HB group and WT+β-HB group, respectively. The malnutrition+β-HB group showed increased body weight compared to the malnutrition group (Fig. 2*A*). RNA sequencing of ovaries from all four groups revealed that the ovarian gene expression profile in the malnutrition group was significantly different from that in the WT group (Fig. 1*H*), while expression in the malnutrition+β-HB group was nearly restored to WT levels (Fig. 2*B*). This suggests that β-HB supplementation can reverse the gene expression changes caused by malnutrition. GO analysis further showed that the expression of follicular and oocyte development-related genes, which was reduced in the malnutrition group, was restored in the malnutrition+β-HB group (Fig. 2*C*), indicating that β-HB supplementation can rescue early ovarian development impaired by malnutrition. Based on these GO terms, we selected representative downregulated genes, including *Nobox, Igf1, Foxo3, Bmpr1b, Esr1, Smad4, Bcl2, Kitl,* and *Fshr,* for visualization, and subsequently confirmed by qPCR that their expression changes were consistent with the RNA-seq results (Fig. 2, *D* and *E*). The abovementioned results demonstrated that β-HB supplementation partially restores transcriptional alterations associated with ovarian development, supporting ketone bodies as an important metabolite for early ovarian development.

**Figure 2:**
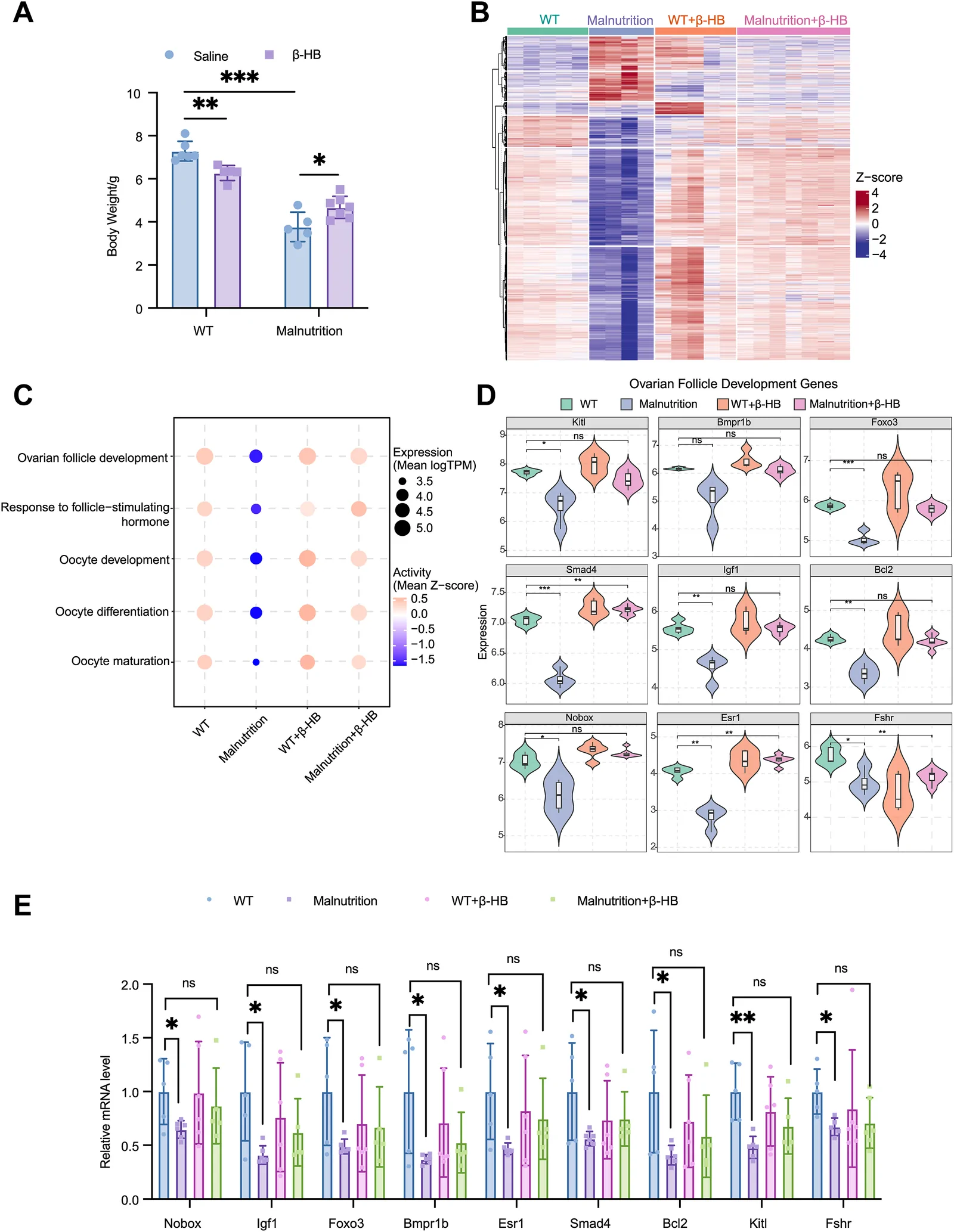
**Ketone body supplementation in neonatal mice during early ovarian development.***A*, body weight of WT, malnutrition, WT+β-HB and malnutrition+β-HB neonatal mice. *B*, RNA sequencing analysis of ovaries from WT, malnutrition, WT+β-HB and malnutrition+β-HB groups. *C*, gene Ontology enrichment analysis of genes associated with follicular development and oocyte development. *D* and *E*, RNA-seq heatmap visualization and qPCR validation of selected genes involved in early ovarian development (*Nobox, Igf1, Foxo3, Bmpr1b, Esr1, Smad4, Bcl2, Kitl* and *Fshr*). All data are presented as the mean ± S.D. ∗*p* < 0.05, ∗∗*p* < 0.01, ∗∗∗*p* < 0.001, ns, no significance. *A*, WT (n = 6), malnutrition (n = 5), WT+β-HB (n = 5) and malnutrition+β-HB (n = 7). *B–D*, WT (n = 5), malnutrition (n = 4), WT+β-HB (n = 5) and malnutrition+β-HB (n = 7). *E*, WT (n = 5), malnutrition (n = 6), WT+β-HB (n = 6) and malnutrition+β-HB (n = 5).

### Neonatal ketone body deficiency impairs adult ovarian function

To better investigate the impact of neonatal ketone body metabolism on ovarian development throughout the female reproductive lifespan, we constructed systemic Hmgcs2 knockout (KO) mice. We first measured HPO-axis hormone levels at the onset of sexual maturity in 8-week-old mice. The results showed that serum FSH, LH, E2, and progesterone were all lower in ketone body–deficient mice than those in controls, indicating impaired adult ovary function (Fig. 3, *A*–*D*). Although the ovarian index did not differ significantly between KO mice and WT mice (Fig. 3, *E*–*G*), the number of primordial follicles was markedly reduced (Fig. 3, *F* and *G*). TUNEL staining also revealed a higher number of apoptotic follicles in the ovaries of ketone body deficient mice (Fig. 3, *H* and *I*). Collectively, these results indicate that neonatal ketone body deficiency impairs adult ovary function in mice by suppressing HPO-axis hormone production and accelerating follicle loss through increased follicular apoptosis.

**Figure 3:**
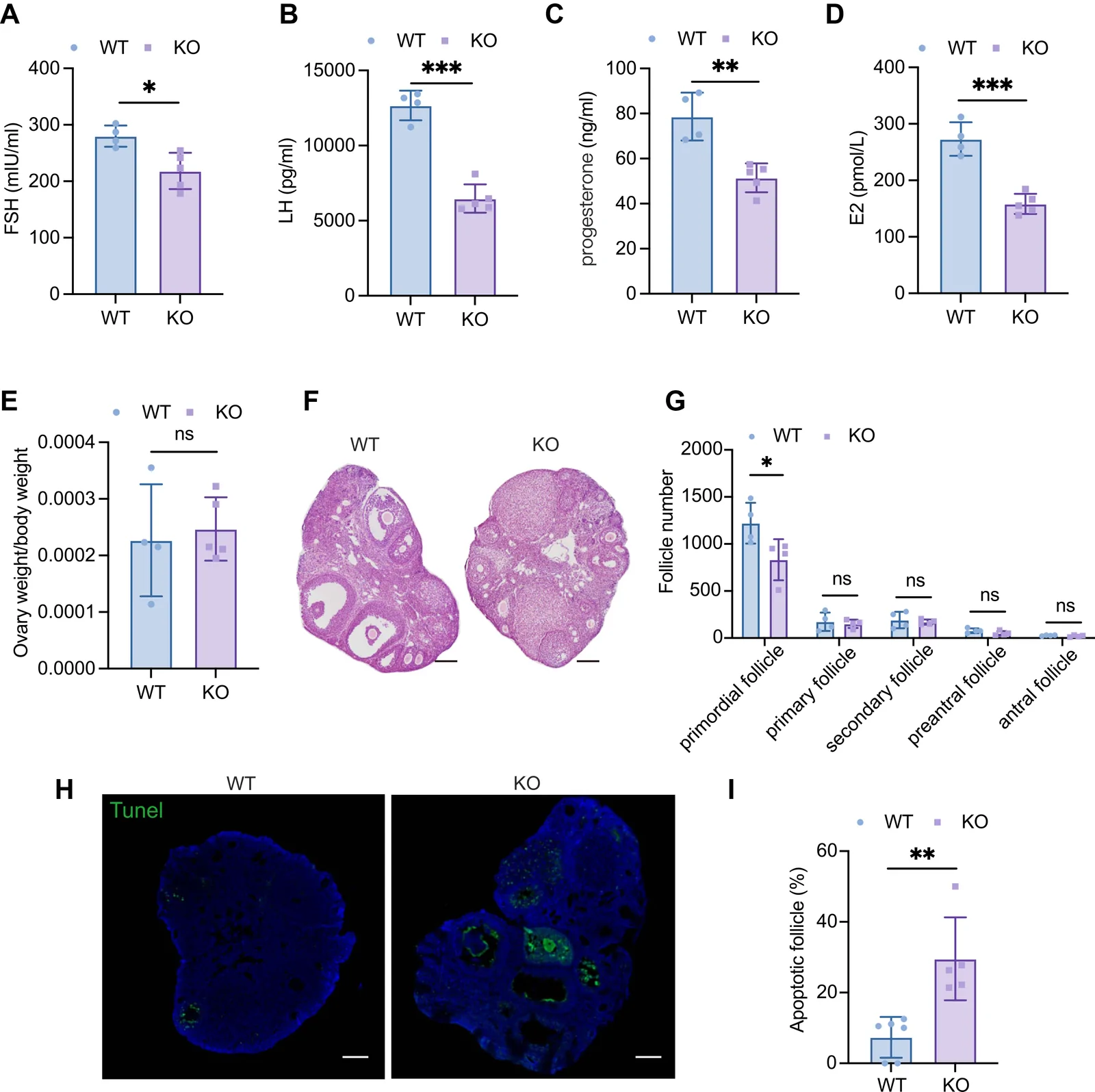
**Neonatal ketone body deficiency impairs adult ovarian function.***A–D*, serum follicle-stimulating hormone (FSH), luteinizing hormone (LH), estradiol (E2) and progesterone levels in 8-week-old WT and systemic Hmgcs2 knockout (KO) mice. *E*, ovarian index in WT and Hmgcs2 KO mice. *F* and *G*, ovarian histology and quantification of follicle numbers in WT and Hmgcs2 KO mice. Scale bar, 100 μm. *H* and *I*, TUNEL staining and quantification of apoptotic follicles in ovaries from WT and Hmgcs2 KO mice. Scale bar, 100 μm. All data are presented as the mean ± S.D. ∗*p* < 0.05, ∗∗*p* < 0.01, ∗∗∗*p* < 0.001, ns, no significance, unpaired *t* test. *A–E*, WT (n = 4), KO (n = 5). *F* and *G*, WT (n = 4), KO (n = 4). *H* and *I*, WT (n = 6), KO (n = 6).

### Adult follicle development and oocyte quality are compromised in neonatal ketone body-deficient mice

Despite the increased follicular apoptosis in ketone body-deficient mice, many follicles still successfully developed to the preovulatory stage. Therefore, we attempted to further investigate the quality of oocytes and ovulation outcomes in adult KO mice. Notably, the adult germinal vesicle (GV) stage oocytes from neonatal ketone body-deficient mice showed no significant difference in the non-surrounded nucleolus to surrounded nucleolus (NSN-SN) transition rate during IVM compared to the WT group, and the germinal vesicle breakdown (GVBD) rate was even higher than that of the WT (Fig. 4, *A*–*C*). However, immunofluorescence staining revealed misaligned chromosomes leading to an expanded equatorial plate in metaphase I (MI) oocytes (Fig. 4, *D* and *E*). In superovulation experiments, KO mice released fewer metaphase II (MII) oocytes, which included abnormal oocytes such as those with fragmentation (Fig. 4, *F*–*H*).

**Figure 4:**
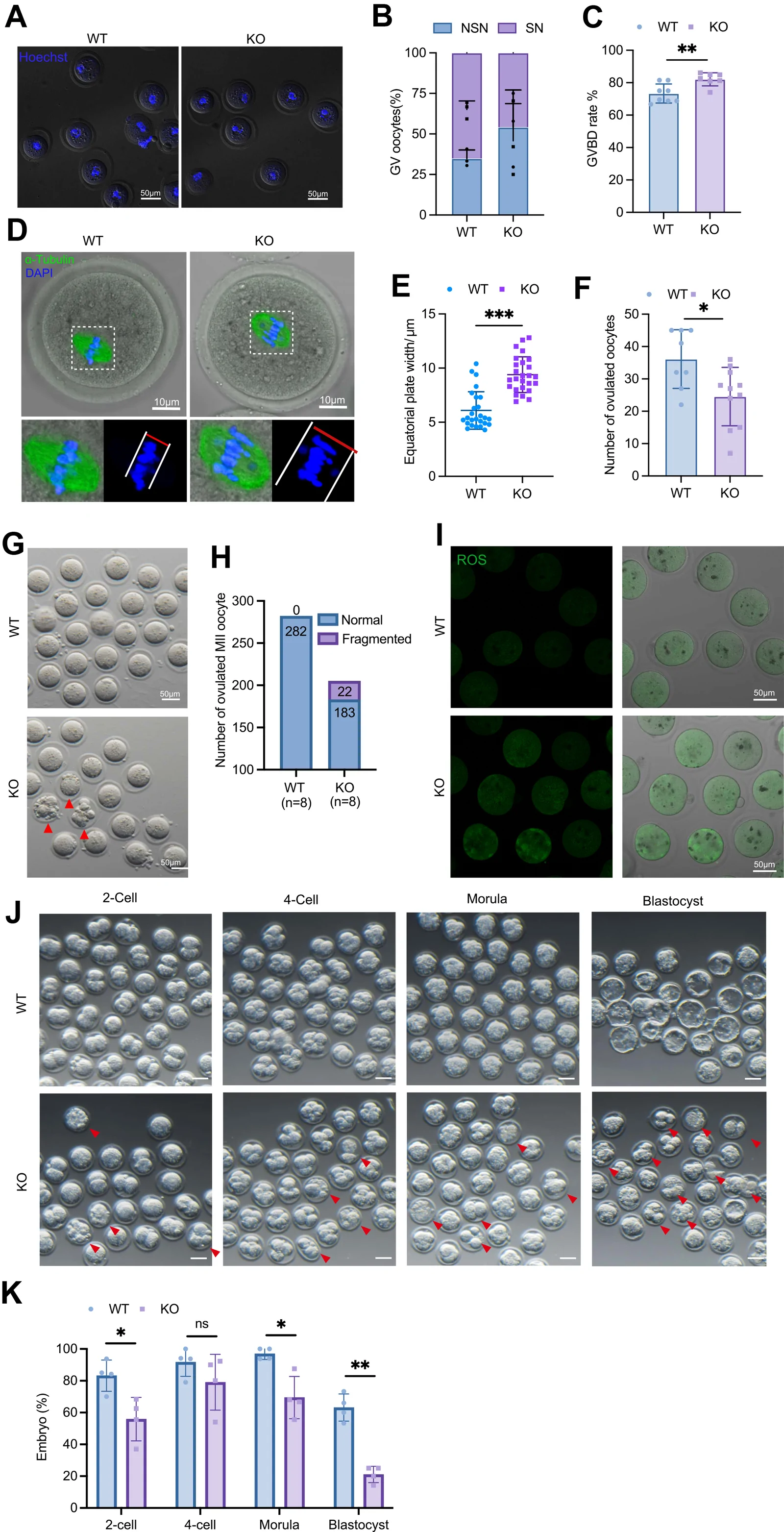
**Adult follicle development and oocyte quality assessment in neonatal ketone body–deficient mice.***A*, immunofluorescence images of germinal vesicle (GV) stage oocytes from WT and KO mice. Scale bar, 50 μm. *B*, quantification of NSN–SN transition in GV stage oocytes. *C*, quantification of germinal vesicle breakdown (GVBD). *D* and *E*, immunofluorescence staining and quantification of chromosome alignment in metaphase I (MI) oocytes from WT and KO mice. Red line, width of the spindle equatorial plate. Scale bar, 10 μm. *F*, quantification of superovulation oocyte numbers. *G*, bright field images of oocytes. *Arrows* indicate fragmented oocytes. Scale bar, 50 μm h, Quantification of fragmented oocytes. *I*, immunofluorescence staining showing ROS levels in oocytes. Scale bar, 50 μm. *J*, *in vitro* fertilization (IVF) and embryo development assessment of morphologically normal MII oocytes. *Arrows* indicate embryos with developmental arrest or degeneration. Scale bar, 50 μm. *K*, embryo formation rates. All data are presented as the mean ± S.D. ∗*p* < 0.05, ∗∗*p* < 0.01, ∗∗∗*p* < 0.001, ns, no significance, unpaired *t* test. *A–E*, WT (n = 8), KO (n = 7). *F*, WT (n = 8), KO (n = 11). *G–I*, WT (n = 8), KO (n = 8). *J* and *K*, WT (n = 4), KO (n = 4).

Previous studies have indicated that excessive accumulation of reactive oxygen species (ROS) can lead to oocyte aging, characterized by a gradual decline in both the quantity and quality of oocytes (17). We assessed the ROS levels in non-fragmented MII stage adult oocytes from neonatal ketone body deficient mice. Despite the absence of visible fragmentation, these oocytes exhibited significantly higher ROS levels compared to WT controls, indicating compromised oocyte quality in KO mice (Fig. 4*I*). Subsequently, we conducted *in vitro* fertilization (IVF) experiments on morphologically normal MII stage oocytes to evaluate their fertilization and embryonic development potential. The results demonstrated that KO oocytes had lower rates of two-cell and morula formation compared to WT, with a blastocyst formation rate of only 20% (Fig. 4, *J* and *K*). Overall, ketone body deficiency leads to reduced oocyte quality, affecting oocyte development, maturation, fertilization, and subsequent embryonic development.

### Ketone bodies regulate oocyte quality by modulating histone acetylation

Protein acetylation is a crucial post-translational modification that plays a key role in oocyte maturation (18). Previous reports have shown that ketogenesis can affect K-ac and K-bhb levels (13). In our study, we first assessed the protein acetylation levels in adult MII stage oocytes and observed a significant increase in acetylation in proteins with a molecular weight of approximately 17 kDa in KO oocytes (Fig. 5*A*), which we suspect corresponds to changes in histone acetylation levels. Consequently, we examined histone H3 acetylation and confirmed its elevation in adult KO oocytes (Fig. 5*B*). To avoid a superficial analysis and pinpoint the specific epigenetic alterations, we further evaluated the acetylation status of specific histone H3 lysine residues. Western blot analysis revealed that the levels of H3K9ac and H3K27ac, two well-established epigenetic marks associated with transcriptionally active chromatin (19, 20), were significantly upregulated in KO oocytes compared to WT controls (Fig. 5*B*). This specific hyperacetylation suggests a more open chromatin state in ketone-deficient oocytes. We then confirmed the function of histone acetylation on oocyte maturation by Trichostatin A (TSA) administration (Fig. 5*C*), a histone deacetylase (HDAC) inhibitor to enhance histone acetylation. We found that the TSA-treated group remained arrested at the GV stage compared to the control group (Fig. 5, *D* and *E*), indicating that enhanced histone acetylation by ketone deficiency could impair oocyte quality.

**Figure 5:**
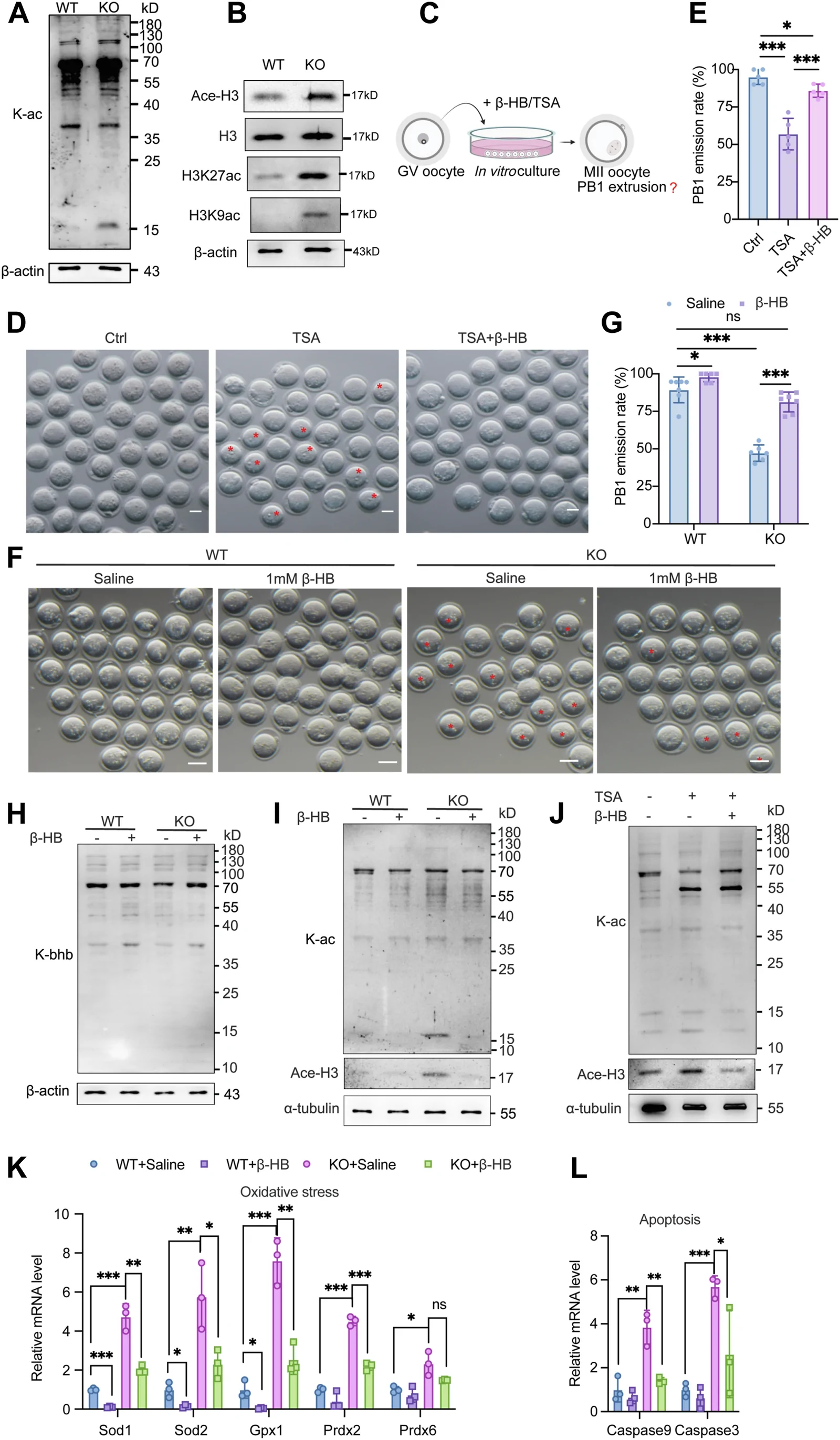
**Analysis of histone acetylation and oocyte maturation in ketone body–deficient oocytes.***A*, Western blot analysis showing protein acetylation (K-ac) levels in adult MII oocytes from WT and KO mice, with a prominent signal at ∼17 kDa. *B*, Western blot analysis showing histone H3, H3 acetylation, H3K27ac, H3K9ac, and β-actin levels in adult MII oocytes from WT and KO mice. *C*, schematic of *in vitro* maturation (IVM) with TSA treatment and/or β-HB supplementation. *D* and *E*, representative images and quantification of GV-stage arrest after TSA treatment, with or without β-HB supplementation. *Asterisk*, oocytes arrested at the GV stage. Scale bar, 50 μm. *F* and *G*, representative images and quantification of first polar body extrusion rates in WT and KO oocytes cultured with or without β-HB. *Asterisk*, oocytes without first polar body extrusion. Scale bar, 50 μm. *H* and *I*, Western blot analysis showing protein lysine β-hydroxybutyrylation (K-bhb) levels and K-ac levels in KO oocytes with or without β-HB supplementation. *J*, Western blot analysis showing K-ac and H3 acetylation levels in TSA-treated oocytes with or without β-HB supplementation. *K* and *L*, RT-qPCR analysis of oxidative stress-related genes (*Sod1、Sod2、Gpx1、Prdx2、Prdx6*) and apoptosis-related genes (*caspase9, caspase3*) in WT and KO oocytes cultured with or without β-HB. All data are presented as the mean ± S.D. ∗*p* < 0.05, ∗∗*p* < 0.01, ∗∗∗*p* < 0.001, ns, no significance. *A* and *B*, WT (n = 5), KO (n = 5). *D, E*, and*, J* Ctrl (n = 5), TSA (n = 5), TSA+β-HB (n = 5). *F–I, K*, and *L*, WT + saline (n = 3), KO + saline (n = 3), WT+β-HB (n = 3), KO+β-HB (n = 3).

To investigate the role of ketone bodies in regulating histone acetylation levels in oocytes, we checked oocyte maturation by adding β-HB to the M2 medium (Fig. 5*C*). We found that KO mouse oocytes exhibited a significantly lower first polar body extrusion rate compared to WT. However, after β-HB supplementation, the extrusion rate of KO oocytes significantly increased, approaching WT levels (Fig. 5, *F* and *G*). Previous studies have shown that exogenous β-HB can induce increased K-bhb of various proteins, and our results confirmed that β-HB supplementation elevated K-bhb levels in KO oocyte proteins (21) (Fig. 5*H*). Notably, the addition of ketone bodies reduced histone acetylation levels of histones in KO oocytes, suggesting that ketone bodies can correct the dysregulation of acetylation levels (Fig. 5*I*). In addition, β-HB could rescue TSA-induced oocyte maturation arrest (Fig. 5, *D* and *E*) and even reduce TSA-induced histone acetylation elevation (Fig. 5*J*).

To further characterize the molecular consequences of ketone body deficiency and β-HB supplementation, we examined the expression of representative oxidative stress- and apoptosis-related genes in oocytes by RT-qPCR. The oxidative stress-related genes *Sod1, Sod2, Gpx1, Prdx2,* and *Prdx6*, as well as the apoptosis-related genes *Casp9* and *Casp3*, were significantly upregulated in KO + Saline oocytes compared with WT + Saline oocytes. β-HB supplementation significantly reduced the expression of *Sod1, Sod2, Gpx1, Prdx2, Casp9*, and *Casp3* in KO oocytes, whereas *Prdx6* showed a decreasing trend that did not reach statistical significance (Fig. 5, *K* and *L*). These results indicate that β-HB largely normalizes stress- and apoptosis-related transcriptional responses induced by Hmgcs2 deficiency.

Collectively, the above results demonstrate that neonatal ketone body deficiency results in compromised adult oocyte quality through altered histone acetylation homeostasis, and β-HB administration could effectively alleviate these defects. This also highlights the critical regulatory role of β-HB oocyte meiotic maturation.

### Ketone body deficiency disrupts histone H3 acetylation homeostasis in oocytes

To investigate whether Hmgcs2 deficiency affects the chromatin landscape of oocytes, we performed genome-wide Ace-H3 CUT&Tag analysis in WT + Saline, WT+β-HB, KO + Saline, and KO+β-HB oocytes. Global heatmap analysis showed that KO + Saline oocytes exhibited increased Ace-H3 signals compared with WT + Saline oocytes. β-HB supplementation partially reduced the elevated Ace-H3 signals in KO oocytes (Fig. 6*A*), suggesting that Hmgcs2 deficiency disrupts histone H3 acetylation homeostasis.

**Figure 6:**
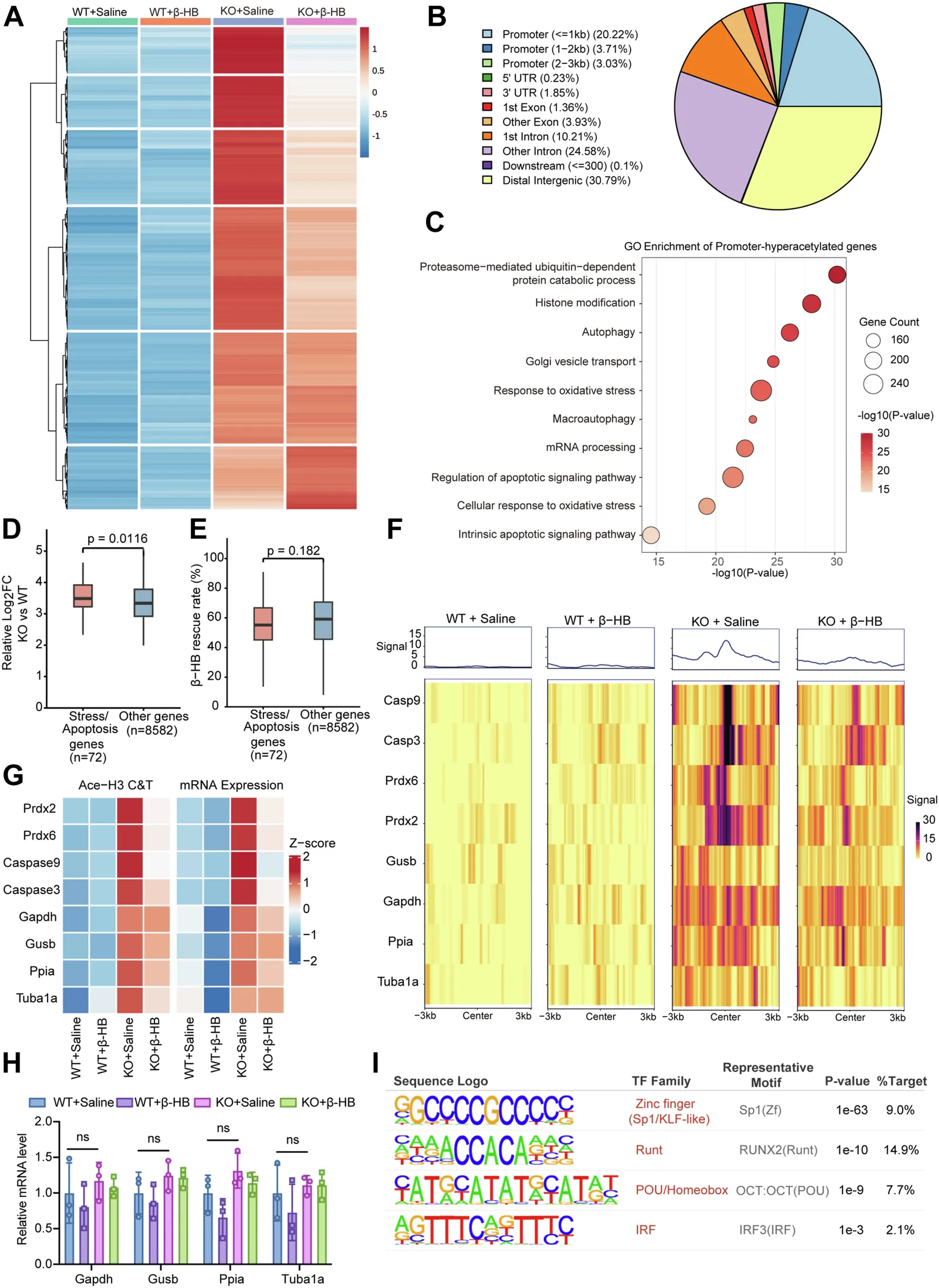
**Ketone body deficieny induces aberrant Ace-H3 enrichment associated with stress-related transcriptional dysregulation in oocytes.***A*, genome-wide heatmap showing Ace-H3 CUT&Tag signal intensity across WT + Saline, WT+β-HB, KO + Saline, and KO+β-HB oocytes. *B*, genomic distribution of Ace-H3 CUT&Tag peaks. *C*, gene ontology (GO) Biological Process enrichment analysis of genes associated with KO-increased promoter Ace-H3 peaks. D, Box plot showing the relative Log2 FC (KO vs WT) of 72 stress/apoptosis-related peaks *versus* other differential promoter peaks (n = 8582); *p* = 0.0116 (Wilcoxon test). *E*, box plot showing the β-HB rescue rate of promoter ace-H3 in the same two groups; *p* = 0.182. *F*, per-gene Ace-H3 signal heatmaps at TSS ± 3 kb for the eight genes analyzed (signal capped at 30). *G*, integrated heatmap combining RT-qPCR-validated mRNA expression changes with Ace-H3 CUT&Tag signals at differential Ace-H3-enriched regions. Values are shown as row-scaled Z-scores. *H*, RT-qPCR analysis of four reference genes (*Gusb, Ppia, Gapdh, Tuba1a*) in WT and KO oocytes cultured with or without β-HB. *I*, top four known motif families enriched in KO-increased promoter-associated Ace-H3 regions identified by HOMER, including zinc finger Sp1/KLF-like, Runt, POU/Homeobox, and IRF motifs. HOMER, Hypergeometric Optimization of Motif EnRichment. All data are presented as the mean ± S.D. WT + Saline (n = 2), KO + Saline (n = 2), WT+β-HB (n = 2), KO+β-HB (n = 2).

Differential Ace-H3 peaks were identified across different genomic regions, including promoters, exons, introns, untranslated regions, and distal intergenic regions (Fig. 6*B*). Since promoter-associated histone acetylation is closely related to gene regulation, we further analyzed genes associated with promoter-localized differential Ace-H3 peaks (22, 23). GO enrichment analysis showed that these genes were mainly involved in oxidative stress response, apoptotic signaling, autophagy, proteasome-mediated protein degradation, and histone modification pathways (Fig. 6*C*). Consistently, stress- and apoptosis-related genes exhibited greater promoter-associated Ace-H3 increases than other gene categories in KO oocytes, whereas the reduction of Ace-H3 signals following β-HB supplementation was comparable between these gene categories (Figure 6, *D* and *E*).

Based on these results, we selected four representative genes related to oxidative stress and apoptosis, including *Prdx2, Prdx6, Casp3, and Casp9*, for further analysis. All four genes contained promoter-associated differential Ace-H3 peaks, and *Casp9* additionally showed an intronic Ace-H3 peak (Fig. S1*A*). Genome browser visualization showed increased Ace-H3 signals at these regions in KO oocytes, which were reduced after β-HB supplementation (Fig. S1*B*).

We further examined the genomic distribution patterns of Ace-H3 signals at these representative loci. *Prdx2, Prdx6, Casp3, and Casp9* showed obvious Ace-H3 accumulation near promoter regions in KO oocytes. In contrast, reference genes, including *Gapdh, Gusb, Ppia,* and *Tuba1a,* showed relatively weak promoter-associated enrichment (Fig. S2). These results suggest that genes involved in oxidative stress and apoptosis are more sensitive to Ace-H3 changes following Hmgcs2 deficiency.

To further determine the distribution pattern of promoter-associated Ace-H3 changes, we performed transcription start site (TSS)-centered analysis. Ace-H3 signals of *Prdx2, Prdx6, Casp3,* and *Casp9* were mainly enriched around the TSS regions and showed higher levels compared with reference genes. In contrast, reference genes displayed more dispersed Ace-H3 signals within the ±3 kb regions surrounding the TSS (Fig. 6*F*).

We next integrated promoter Ace-H3 signals with gene expression changes to explore their relationship with transcriptional regulation. Heatmap analysis based on promoter ±3 kb regions showed that increased Ace-H3 enrichment at *Prdx2, Prdx6, Casp3,* and *Casp9* was accompanied by elevated mRNA expression in KO + Saline oocytes (Figs. 5, *K* and *L* and 6*G*). Reference genes exhibited weaker promoter Ace-H3 enrichment, with no significant alterations in transcript abundance across experimental groups (Fig. 6, *G* and *H*). These findings suggest that promoter-associated Ace-H3 accumulation is preferentially associated with transcriptional activation of stress- and apoptosis-related genes rather than causing a global increase in gene transcription.

To identify potential regulatory features associated with promoter Ace-H3 changes, we performed motif enrichment analysis using promoter-localized differential Ace-H3 peaks. Several transcription factor motifs, including Sp1/KLF-like zinc finger, Runt, POU/Homeobox, and IRF motifs, were significantly enriched. Among them, Sp1/KLF-like motifs showed the strongest enrichment (Fig. 6*I*). These motifs were also detected near the transcription start sites of Prdx2, Prdx6, Casp3, and Casp9 (Fig. S1, *C* and *D*). These results suggest that Sp1/KLF-related motifs may be associated with promoter regions undergoing Ace-H3 alterations after Hmgcs2 deficiency.

Together, these findings indicate that Hmgcs2 deficiency causes widespread changes in Ace-H3 distribution and disrupts histone H3 acetylation homeostasis in oocytes. However, these alterations are not evenly distributed across genes. Oxidative stress- and apoptosis-related genes show preferential promoter-associated Ace-H3 enrichment accompanied by increased transcriptional changes. β-HB supplementation partially restores the abnormal Ace-H3 landscape, suggesting that ketone body metabolism contributes to the maintenance of oocyte chromatin homeostasis.

### Adult oocyte defects induced by lactational malnutrition are partially rescued by β-HB

Neonatal ketone body deficiency causes adult abnormal hormone profiles, reduced oocyte quality, and impaired embryo development. We therefore tested whether lactational malnutrition similarly affects ovarian function and oocyte quality in adulthood. Mice were subjected to undernutrition for 3 weeks during lactation and then refed a normal diet for 5 weeks. After refeeding, body weight returned to WT levels (Fig. 7*A*). Notably, at 8 weeks of age, LH, FSH, and E2 levels were lower than in WT mice (Fig. 7, *B*–*E*) like neonatal ketone body-deficient mice (Fig. 3, *A*–*D*), whereas progesterone was unchanged, indicating ovary dysfunction despite nutritional recovery. Then, we found malnourished mice had a reduced number of ovulated MII-stage oocytes, with occasional occurrences of a few fragmented oocytes (Fig. 7, *F* and *G*). *In vitro* fertilization experiments on superovulated MII oocytes showed a significantly lower two-cell rate compared to WT groups, with almost no blastocyst formation (Fig. 7, *H* and *I*). These findings suggested that lactational malnutrition leads to persistent defects in oocyte developmental competence till adulthood. Considering the role of β-HB on neonatal ketone body deficient oocyte, we therefore investigated whether supplementing β-HB during *in vitro* maturation of GV-stage oocytes could ameliorate this impairment. The results turned out that β-HB supplementation substantially improved oocyte quality, reflected by fewer fragmented oocytes in the malnutrition+β-HB group than in the malnutrition + saline group (Fig. 7, *J* and *K*). Overall, these results indicate that lactational malnutrition induces impairments in adult ovarian function and oocyte quality, and β-HB supplementation could partially rescue compromised oocyte quality.

**Figure 7:**
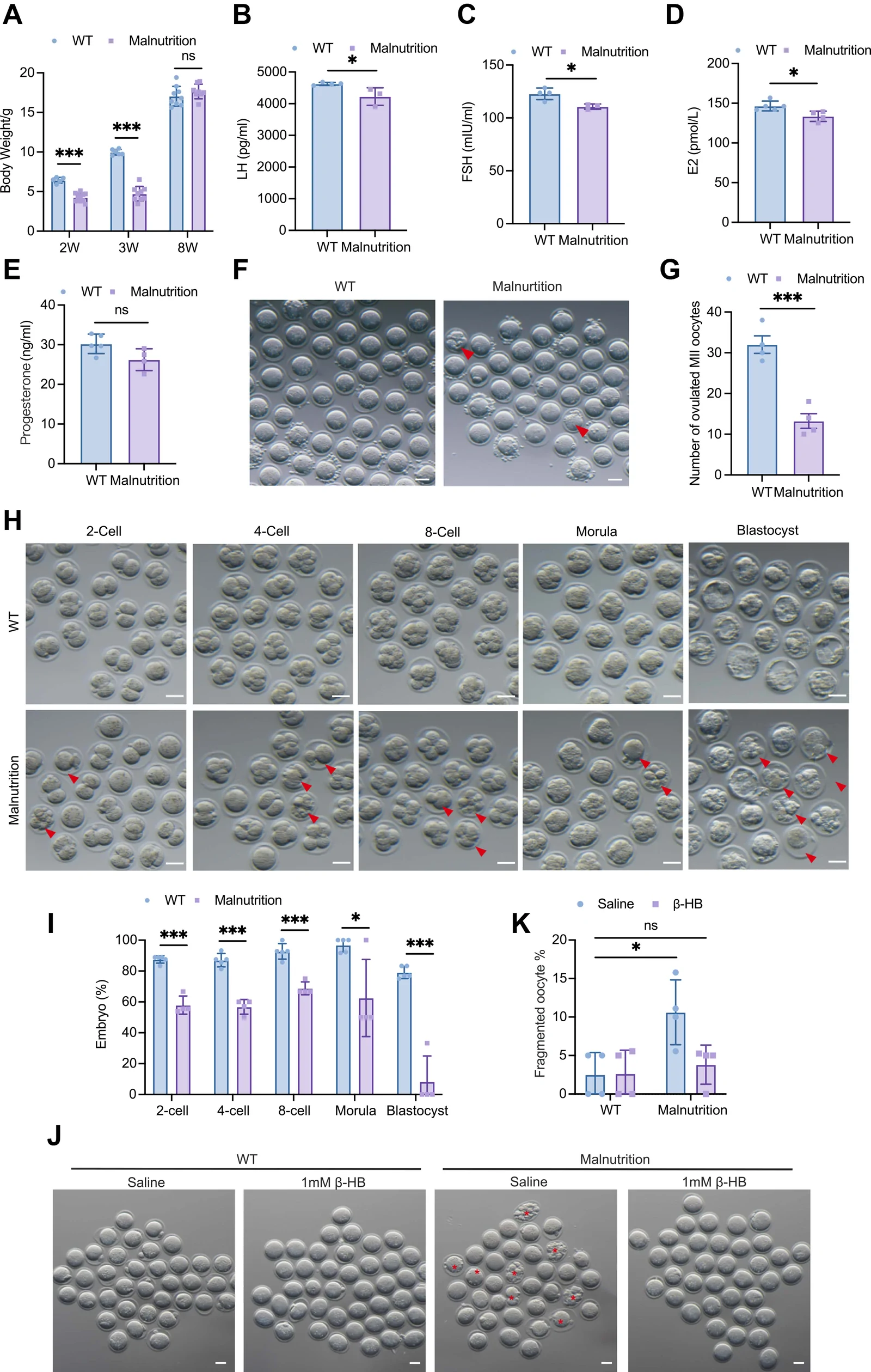
**Adult ovarian function and oocyte competence after lactational undernutrition and β-hydroxybutyrate supplementation.***A*, body weight of WT and malnutrition mice at 2 weeks, 3 weeks, and 8 weeks after recovery on a standard chow diet. *B–E*, serum levels of LH, FSH, E2, and progesterone in adult WT and Malnutrition mice. *F* and *G*, representative images and quantification of ovulated MII-stage oocytes from WT and Malnutrition mice, with fragmented oocytes indicated. Arrows indicate fragmented oocytes. Scale bar, 50 μm. *H* and *Iin vitro* fertilization outcomes of superovulated MII oocytes from WT and Malnutrition mice, showing two-cell embryo rates and blastocyst formation efficiency. *Arrows* indicate embryos with developmental arrest or degeneration. Scale bar, 50 μm. *J* and *K*, representative images and quantification of oocytes after *in vitro* maturation (IVM) of GV-stage oocytes from Malnutrition mice cultured with saline or β-hydroxybutyrate (β-HB). *Asterisks* indicate fragmented oocytes. Scale bar, 50 μm. All data are presented as the mean ± S.D. ∗*p* < 0.05, ∗∗*p* < 0.01, ∗∗∗*p* < 0.001, ns, no significance, unpaired *t* test. *A*, WT (n = 5), Malnutrition (n = 9). *B* and *C*, WT (n = 4), Malnutrition (n = 3). *D* and *E*, WT (n = 5), Malnutrition (n = 4). *F* and *G*, WT (n = 4), Malnutrition (n = 4). *H* and *I*, WT (n = 5), Malnutrition (n = 4). *J* and *K*, WT (n = 4), Malnutrition (n = 4).

### Neonatal ketone body-deficient female mice are susceptible to secondary nutritional stress

Our previous research indicated that the number of offspring produced by 8-week-old KO mice did not differ significantly from that of WT mice. However, a marked decline in fertility was observed after 14 weeks of age (15). In modern life, women often face the risk of overnutrition stress during adulthood. We then investigated the effects of high-fat diet (HFD) overnutrition exposure during early reproductive life on oocyte quality in neonatal ketone body deficient mice. A short 1-week of HFD feeding could increase the ovarian index in ketone body deficiency mice (Fig. 8*A*). However, no significant differences in HPO-axis hormone levels were observed between control diet (CD) or HFD exposure mice (Fig. 8, *B*–*E*). Reproductive ageing leads to increased collagen deposition and accumulation of multinucleated giant cells (MNGCs) in the ovary (24, 25). HE staining of ovarian sections revealed that KO mice subjected to 1 week of HFD exhibited significantly larger areas of MNGC plaques compared to the WT group (Fig. 8, *F* and *G*). This finding suggests that HFD feeding accelerates reproductive aging in ketone body-deficient mice. Similarly, IVF experiments conducted on MII oocytes from mice subjected to 1 week of HFD revealed that the HFD-KO group exhibited significantly reduced fertilization rates and impaired embryonic development (Fig. 8, *H* and *I*). The findings above strongly indicate that neonatal ketone body deficient mice not only exhibit compromised adult oocyte quality but are also susceptible to overnutritional stress when faced with a secondary nutritional challenge, thereby accelerating reproductive aging.

**Figure 8:**
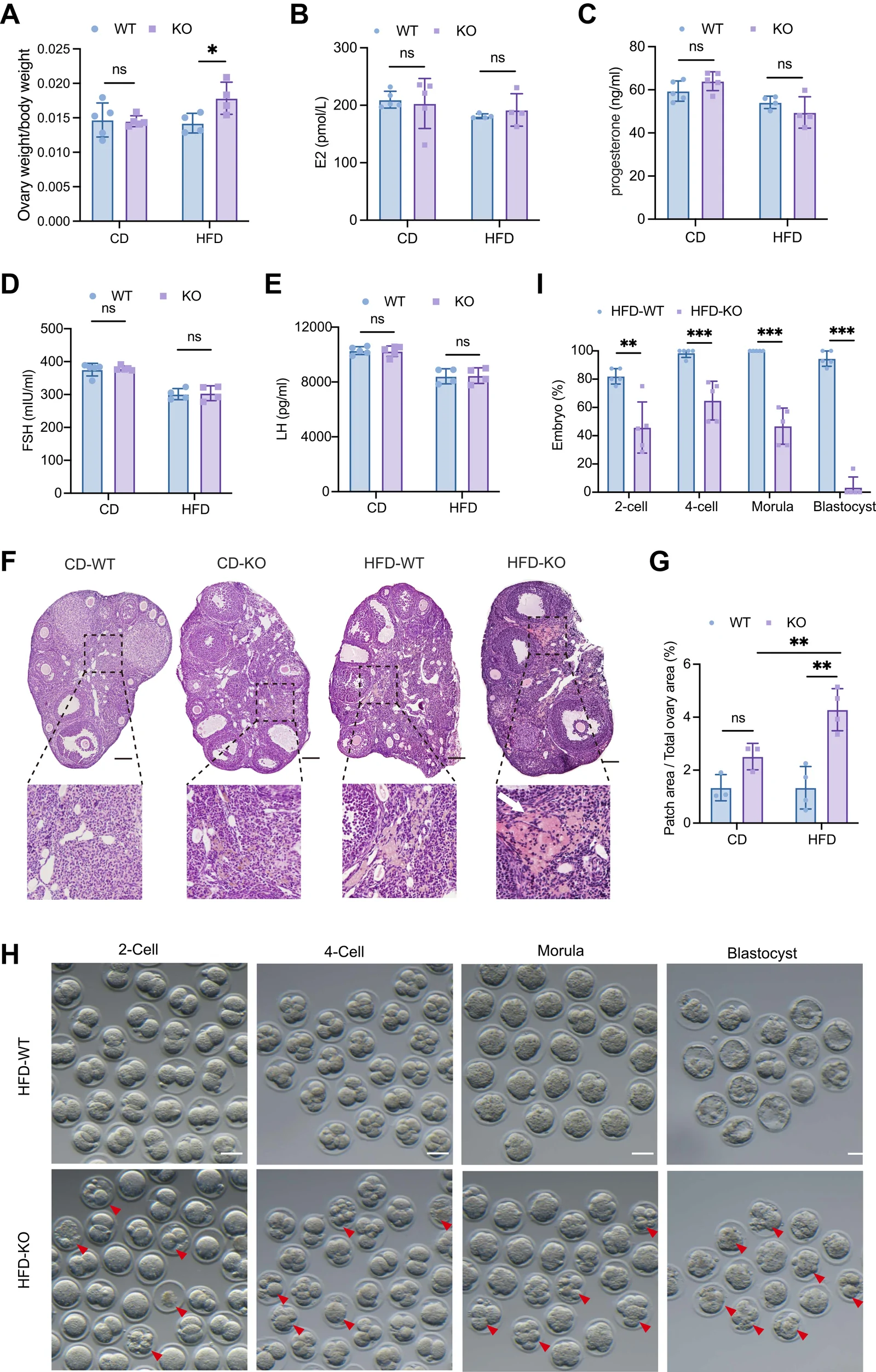
**Effects of a 1-week HFD on reproductive function in ketone body-deficient female mice.***A*, ovarian index in KO and WT mice after 1 week of high-fat diet (HFD) exposure. *B–E*, serum levels of HPO-axis hormones (LH, FSH, E2, progesterone) in KO and WT mice after 1 week of HFD or control diet (CD) exposure. *F* and *G*, representative images and quantification of MNGC plaques in ovarian sections from KO and WT mice exposed to 1 week of HFD. Scale bar, 100 μm. *H* and *I*, *in vitro* fertilization outcomes of MII oocytes from KO and WT mice subjected to 1 week of HFD exposure, showing fertilization rates and embryonic development. *Arrows* indicate embryos with developmental arrest or degeneration. Scale bar, 50 μm. All data are presented as the mean ± S.D. ∗*p* < 0.05, ∗∗*p* < 0.01, ∗∗∗*p* < 0.001, ns, no significance, unpaired *t* test. *A–E*, CD-WT (n = 5), HFD-WT (n = 5), CD-KO (n = 4), HFD-KO (n = 4). *F* and *G*, CD-WT (n = 3), HFD-WT (n = 3), CD-KO (n = 3), HFD-KO (n = 3). *H* and *I*, HFD-WT (n = 5), HFD-KO (n = 5).

## Discussion

Early-life nutrition plays a pivotal role in shaping organ development and long-term functional capacity, consistent with the developmental origins framework in which transient nutritional exposures durably influence disease risk later in life (26, 27, 28). Although most evidence has focused on metabolic organs, emerging work suggests that the ovary is also vulnerable to early nutritional cues, with perinatal undernutrition or overnutrition linked to altered folliculogenesis, disrupted endocrine homeostasis, and shortened reproductive lifespan in animal models (29, 30). The neonatal period is characterized by a physiological surge in circulating ketone bodies driven by milk lipid utilization (31), and ketone bodies are increasingly recognized as signaling metabolites capable of regulating chromatin state in multiple tissues (32, 33, 34). Building on these concepts, our study identifies this neonatal high-ketone phase as a critical programming window for female reproduction and implicates ketone availability as an important determinant of long-term oocyte quality and reproductive outcomes. Although lactational malnutrition affects multiple metabolic pathways, the combination of transcriptomic discovery, the postnatal ketone surge coinciding with follicle pool establishment, β-HB-rescue experiments, and genetic knockout validation supports a model in which ketone body deficiency serves as a key metabolic link between early nutrition and reproductive programming.

Consistent with previous studies indicating that nutritional status affects the HPO axis and follicle development (35, 36, 37), our data reveal that transient neonatal ketogenesis is required for the establishment of the initial follicle pool and long-term reproductive capacity. By combining lactational nutritional manipulation, β-HB supplementation, and Hmgcs2 genetic deficiency, our study further demonstrates that ketone availability is a key determinant of oocyte quality in adulthood.

Functionally, ketone body deficiency resulted in persistent defects in ovarian function and oocyte developmental competence. Hmgcs2-deficient females showed reduced oocyte quality, compromised meiotic maturation, and impaired early embryonic development. These reproductive defects were accompanied by increased oxidative stress and apoptosis-related changes, indicating that insufficient ketone availability during early life has long-lasting consequences for female reproductive capacity. Together with the β-HB rescue results, these phenotypes support a causal role of ketone body deficiency in linking early nutritional insufficiency to durable oocyte dysfunction.

One point that should be considered is that the increased Ace-H3 levels observed in P7 malnourished ovaries do not necessarily contradict the broad transcriptional down-regulation detected by RNA-seq. Although histone acetylation is generally associated with active transcription at specific promoters and enhancers, global histone H3 hyperacetylation does not uniformly lead to global gene activation. Transcriptional output is shaped by the genomic distribution of acetylation marks, local chromatin context, transcription factor availability, chromatin accessibility, and metabolic state (38, 39, 40). In malnourished ovaries, reduced ketone body metabolism, endocrine changes, oxidative stress, impaired follicle development, and altered cellular composition may collectively suppress gene expression despite increased global Ace-H3 levels. Thus, elevated Ace-H3 in neonatal malnourished ovaries likely reflects an aberrant chromatin acetylation state associated with developmental and metabolic stress, rather than a simple marker of global.

Ketone body deficiency also altered histone acetylation patterns in oocytes. Western blot analysis showed increased H3K9ac and H3K27ac levels in Hmgcs2-deficient oocytes. Consistently, genome-wide Ace-H3 CUT&Tag analysis revealed an overall increase in histone H3 acetylation signals in KO oocytes. Since H3K9ac, H3K27ac, and Ace-H3 are commonly associated with active or accessible chromatin states, these results indicate that ketone body deficiency affects the normal acetylation landscape in oocytes (41, 42, 43). However, similar to the findings in malnourished ovaries, increased global histone acetylation does not necessarily indicate overall transcriptional activation. Instead, the biological effects of these acetylation changes may depend on their genomic locations, local chromatin environment, transcription factor activity, and other regulatory mechanisms.

The CUT&Tag analysis further showed that these acetylation changes were not uniformly distributed across the genome. Differential Ace-H3 peaks were detected in both promoter and non-promoter regions, suggesting that ketone body deficiency causes widespread but diverse chromatin alterations. Because promoter-associated histone acetylation is more closely related to transcriptional regulation, we further analyzed promoter-associated differential peaks (22, 23). Genes associated with these peaks were mainly enriched in pathways related to oxidative stress response, apoptotic signaling, autophagy, proteasome-mediated protein degradation, and histone modification. Compared with reference genes, stress- and apoptosis-related genes showed more pronounced promoter-associated Ace-H3 enrichment in KO oocytes, suggesting that acetylation changes were more evident in stress-related gene groups rather than occurring equally across all genes. These genes also showed greater transcriptional changes than reference genes, and β-HB supplementation partially reversed both Ace-H3 enrichment and gene expression alterations. Nevertheless, Ace-H3 enrichment alone could not fully predict transcriptional changes at every locus, indicating that histone acetylation represents one regulatory layer among multiple mechanisms controlling gene expression. Together, these findings suggest that ketone body deficiency induces chromatin alterations that are preferentially associated with stress-related genes and may contribute to transcriptional dysregulation. Motif enrichment analysis further identified potential regulatory elements within promoter-associated differential Ace-H3 regions, with the Sp1/KLF-like zinc finger motif showing the strongest enrichment. Further studies are needed to determine whether transcription factors recognizing these motifs directly regulate these affected genes.

Early nutritional insults have been reported to increase susceptibility to metabolic diseases and age-related dysfunctions (*e.g.*, obesity, diabetes, and cardiovascular diseases), at least in part through persistent epigenetic alterations in metabolic tissues such as the liver and adipose tissue (44, 45, 46, 47). Similarly, our study demonstrates that ketone-deficient females show heightened vulnerability to secondary nutritional stress (*e.g.*, short-term high-fat diet), extending previous reports of "second-hit" susceptibility following early-life malnutrition. Notably, our findings highlight that this resilience phenotype is also observed in the ovary (26, 48), thereby linking early ketone-dependent epigenetic programming to later-life reproductive robustness. Together, these findings position neonatal ketone bodies as a previously underappreciated developmental signal that connects early nutrition to long-term ovarian function and stress adaptability, providing a mechanistic and temporally defined refinement to the existing literature.

Our study reveals an important role of early ketogenesis in the metabolic programming of female reproduction and expands our understanding of ketone bodies as metabolic and epigenetic regulators of ovarian function. These findings suggest that nutritional status and ketone availability during critical developmental windows may influence long-term ovarian function. Future studies should further determine the effects of ketone-based interventions on ovarian function and oocyte quality and investigate ketone-dependent chromatin remodeling and other related post-translational modifications, such as lysine acetoacetylation, in reproductive programming (49, 50). Together, our findings provide new insights into how ketone metabolism regulates oocyte quality and reproductive lifespan, and may help elucidate the mechanisms underlying early-onset ovarian aging and infertility.

## Experimental procedures

### Animals

All animal procedures were approved by the Animal Care and Use Committee of Nanjing Medical University and adhered to institutional guidelines (Protocol No. IACUC-2004029). C57BL/6 mice were obtained from the Animal Core Facility of Nanjing Medical University. Systemic Hmgcs2 knockout mice were generated by CRISPR/Cas9-mediated genome editing. Mice were housed under a 12-h light/12-h dark cycle at 22 °C with controlled humidity, with ad libitum access to food and water unless otherwise indicated.

For the malnutrition model, female C57BL/6J pups were subjected to litter-size expansion starting at postnatal day 1 (P1), whereby 15 pups were nursed by a single dam throughout the lactation period. After weaning, the pups were returned to a standard chow diet. During ketogenic diet (KD) feeding, mice were injected intraperitoneally with either saline or β-hydroxybutyrate (BHB; 400 mg kg^−1^ day^−1^).

### Oocyte collection and culture

Female mice were superovulated with 5 IU pregnant mare serum gonadotropin (PMSG) followed by 5 IU human chorionic gonadotropin (hCG) 48 h later. Mice were euthanized by cervical dislocation 12 h after hCG injection. GV and GVBD oocytes were collected by rupturing antral follicles and removing cumulus cells by pipetting. MII oocytes were retrieved from the oviductal ampullae, and cumulus masses were removed in medium containing 0.5 mg ml^−1^ hyaluronidase at 37 °C. For *in vitro* maturation, fully grown GV oocytes were cultured in M2 medium (Millipore, Merck) under mineral oil at 37 °C in a 5% CO_2_ incubator.

### Oocyte culture with β-hydroxybutyrate and TSA

GV-stage oocytes were cultured in M2 medium supplemented with 1 mM β-hydroxybutyric acid sodium (MCE, Cat. HY-W010452) or 500 nM trichostatin A (TSA; MCE, Cat. HY-15144) under mineral oil at 37 °C in a 5% CO_2_ incubator until MII stage was reached.

### *In vitro* fertilization and embryo culture

Sperm were isolated from the cauda epididymides of 10–20-week-old C57BL/6 male mice and capacitated in HTF fertilization medium (Millipore, Merck) supplemented with 10 mg ml^−1^ bovine serum albumin (BSA). After fertilization, zygotes were cultured in KSOM medium (Millipore, Merck) under 5% CO_2_, 5% O_2_, and 90% N_2_ at 37 °C.

### Western blotting

Oocytes were pooled and lysed in Laemmli sample buffer with protease inhibitors. The lysates were subjected to SDS–PAGE, and proteins were transferred to PVDF membranes, which were blocked for 1 h in PBS with 0.1% Tween-20 and 5% non-fat milk. Membranes were incubated with primary antibodies, followed by HRP-conjugated secondary antibodies. Protein bands were visualized with Pierce ECL Western Blotting Substrate (Tanon).

### Immunofluorescence

Oocytes were fixed with 4% paraformaldehyde for 30 min and permeabilized with 0.5% Triton X-100 for 20 min. After blocking in 1% BSA in PBS for 1 h, oocytes were incubated overnight at 4 °C with anti-tubulin primary antibody. The secondary antibody was incubated for 2 h at room temperature. Chromosomes were stained with Hoechst 33,342 for 15 min. Samples were mounted with anti-fade medium (Servicebio) and imaged using a laser scanning confocal microscope (LSM 900, Zeiss). Fluorescence intensity and spindle length were quantified using ImageJ.

### Histology and follicle counting

Ovarian tissues were fixed overnight at 4 °C in 4% paraformaldehyde, dehydrated through ethanol, embedded in paraffin, and sectioned at 5 μm. Sections were stained with haematoxylin and eosin (H&E) according to standard protocols. Follicles containing visible oocytes were counted under a high-power microscope, and follicular stages were classified based on established criteria. Follicle and oocyte diameters were measured using a Nikon light microscope.

### TUNEL assay

Apoptotic cells were detected with a DeadEnd Colorimetric TUNEL Assay Kit (Promega). Paraffin-embedded ovarian sections were processed per the manufacturer’s instructions and imaged using an LSM 900 confocal microscope (Zeiss). Adjacent sections were stained with H&E for total follicle count. The proportion of apoptotic follicles was determined by the ratio of TUNEL-positive follicles to total follicles.

### Measurement of serum β-hydroxybutyrate and reproductive hormones

Serum β-hydroxybutyrate levels were measured using a β-Hydroxybutyrate Colorimetric Assay Kit (BioVision, Cat. K632-100). Serum concentrations of luteinizing hormone (LH), follicle-stimulating hormone (FSH), estradiol (E2), and progesterone were measured using ELISA kits (Shanghai Enzyme-linked Biotechnology; Cat. ml063366, ml001910 M, ml063198, ml058395).

### ROS measurement

Intracellular ROS levels were assessed using a ROS Assay Kit (Beyotime, Cat. S0033S). Oocytes were incubated in 10 μM DCFH-DA for 30 min at 37 °C, then washed three times and transferred to live-cell imaging dishes. ROS levels were imaged using a laser scanning confocal microscope (LSM 900, Zeiss).

### RNA isolation and quantitative real-time PCR

Total RNA was extracted from mouse ovaries or oocytes using TRIzol reagent (Takara, 9109), and RNA concentration was measured using a NanoDrop 2000 spectrophotometer (Thermo Fisher Scientific). Reverse transcription was performed using PrimeScript RT Master Mix (Takara) with 1 μg RNA. Quantitative PCR was carried out using ChamQ Universal SYBR qPCR Master Mix (Vazyme, Q711) on a ViiA 7 Real-Time PCR System (Applied Biosystems). Gene expression was normalized to 18S rRNA, and relative mRNA levels were calculated using the 2^-^ΔΔCt method.

### RNA sequencing and bioinformatic analysis

RNA sequencing was conducted by Novogene. mRNA was enriched using Oligo(dT) magnetic beads, fragmented, and reverse-transcribed to generate cDNA. Libraries were prepared *via* end repair, A-tailing, adaptor ligation, size selection, and amplification. Libraries passing quality control were pooled and sequenced on an Illumina platform. Raw reads were processed with fastp to remove adaptor sequences, poly-N stretches, and low-quality reads. Clean reads were quantified using featureCounts (v2.0.6). Differential expression analysis was conducted with DESeq2 (v1.42.0), and *p* values were adjusted using the Benjamini-Hochberg method. Genes with padj < 0.05 and | log_2_ (fold change) | > 1 were considered differentially expressed. Gene Ontology enrichment was analyzed with clusterProfiler (v4.8.1), and gene set enrichment analysis (GSEA) was performed using predefined ketone body metabolism–related gene sets with the GSEA software package.

### CUT&Tag library preparation

CUT&Tag assays were performed using the Hyperactive Universal CUT&Tag Assay Kit for Illumina Pro (Vazyme Biotech Co, Ltd). Briefly, 40 oocytes per sample were cultured in M2 medium to the MII stage. The oocytes were then washed three times with PVA-PBS and resuspended in 100 μl of wash buffer. The oocytes were incubated with activated ConA Beads Pro, followed by supernatant removal and overnight incubation with a primary antibody against Histone H3 (acetyl K9+K14 + K18 + K23 + K27) (1:100; Abcam, ab47915) in antibody buffer at 4 °C. After washing, the oocytes were incubated with a secondary antibody Goat anti-Rabbit IgG (1:100; Vazyme, Ab207–01) at room temperature for 30 to 60 min, followed by supernatant removal and incubation with diluted pA/G-Tnp Pro at room temperature for 1 h. Tagmentation was initiated by adding diluted Trueprep tagment buffer L and incubating at 37 °C for 60 min. DNA was enriched using DNA Extract Beads and amplified for 28 cycles. Paired-end sequencing was performed on the Illumina NovaSeq 6000 platform (Nanjing Jiangbei New Area Biopharmaceutical Public Service Platform).

### CUT&Tag data analysis

Raw sequencing reads were trimmed with fastp (v.0.20.1) and aligned to the mm10 mouse reference genome by Bowtie2 (v.2.2.5) with default parameters. Peaks were called with MACS2 (v.2.1.2; P < 1e-5) against the matched IgG sample, and peaks reproducibly detected in ≥2 biological replicates per condition were retained. RPKM-normalized bigWig tracks were generated with deepTools, and promoter signals were log2-transformed with a pseudocount of 0.5 to avoid undefined values, followed by differential analysis with limma (|log2FC| > log2(5); BH-adjusted *p* < 0.05). Peak annotation to promoters (TSS ± 3 kb) was performed with ChIPseeker and GO Biological Process enrichment was analyzed with clusterProfiler. De novo motif discovery within KO-upregulated promoter peaks was performed with HOMER (findMotifsGenome.pl); motifs detected in the promoter peaks of Prdx2, Prdx6, Casp3 and Casp9 were classified into transcription factor families based on their DNA-binding domains, and their positions relative to the TSS were mapped. For the eight genes analyzed (target genes: Prdx2, Prdx6, Casp9 and Casp3; housekeeping genes: Gapdh, Gusb, Ppia and Tuba1a), per-gene heatmaps (TSS ± 3 kb), gene-body profiles with ± 3 kb flanking regions, and a combined qPCR mRNA/H3K27ac Z-score heatmap (TSS ± 1 kb) were generated. Promoter Ace-H3 changes of 72 stress/apoptosis-related peaks *versus* other differential promoter peaks (n = 8582) were compared with the Wilcoxon rank-sum test, and the BHB rescue rate was calculated as (1 − (KO + β-HB)/(KO + Saline)) × 100%. Genome browser tracks were visualized with IGV, and plots were generated in R using pheatmap and ggplot2.

### Statistical analysis

Data are presented as mean ± SD, with individual data points, each representing a biological replicate, superimposed on the bar graphs. Statistical significance between two groups was determined using an unpaired two-tailed Student’s *t* test, and comparisons between multiple groups were made using two-way ANOVA. A *p* value < 0.05 was considered statistically significant.

## Data, materials, and software availability

The authors declare that all relevant data of this study are available within the article or from the corresponding author (lichaojun@njmu.edu.cn) upon reasonable request.

The RNA-seq and CUT&Tag datasets generated in this study have been deposited in the Gene Expression Omnibus (GEO) database under accession codes GSE341980 (RNA-seq) and GSE342089 (CUT&Tag).

## Supporting information

This article contains supporting information.

## Conflict of interest

The authors declare that they have no conflicts of interest with the contents of this article.
