# Supplementary material for: Postnatal ketone deficiency impairs adult oocyte quality via enhancing histone acetylation in malnourished mice
Source: J Biol Chem. 2026 Sep 8;302(10):113535. doi: 10.1016/j.jbc.2026.113535 (PMC13671086; doi:10.1016/j.jbc.2026.113535)
Supplement: Supplementary file 1 [file mmc1.pdf]

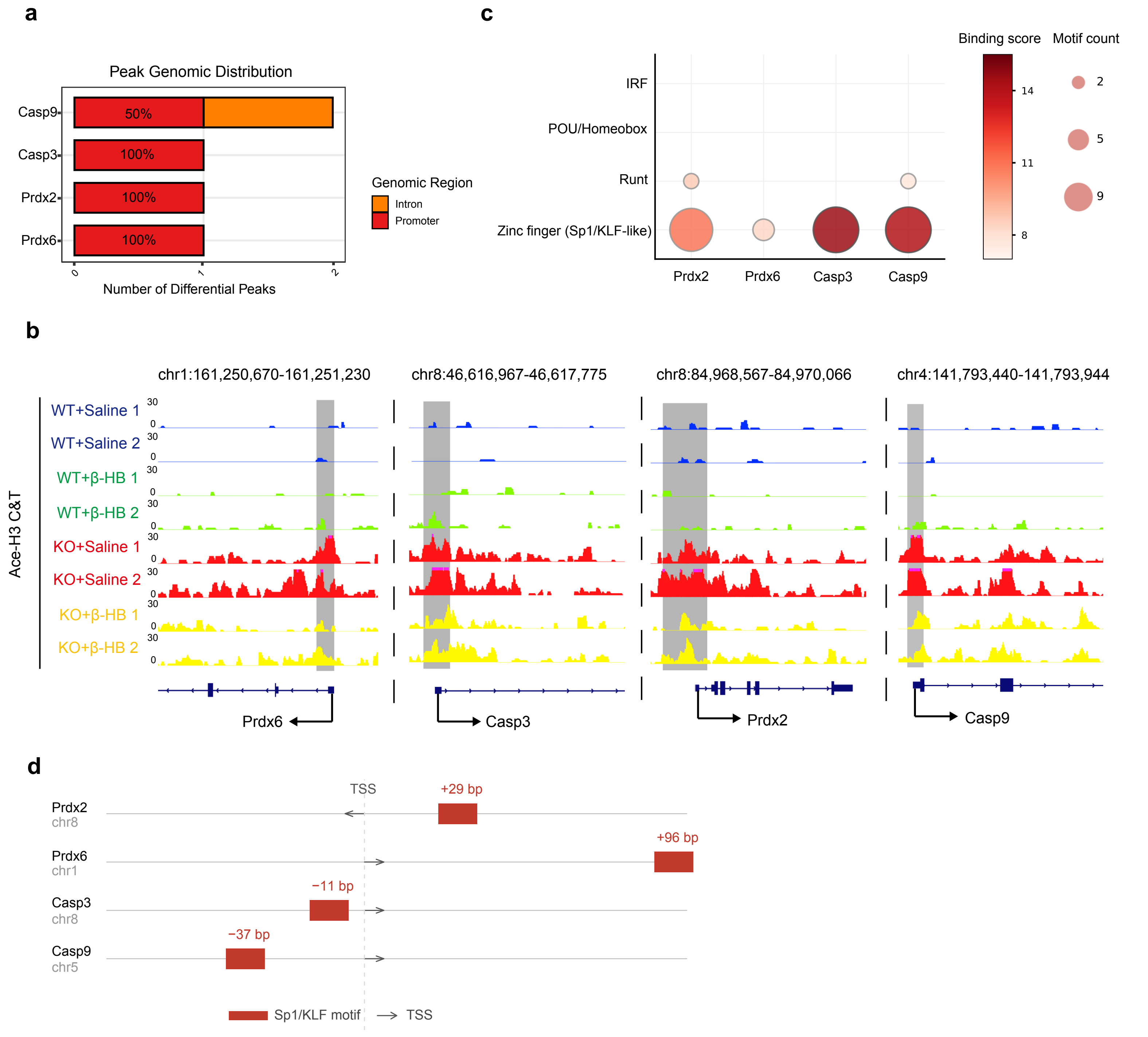

a
Peak Genomic Distribution
Casp9
Casp3
Prdx2
Prdx6
50%
100%
100%
100%
0
1
2
Number of Differential Peaks
Genomic Region
Intron
Promoter
c
Binding score
Motif count
IRF
POU/Homeobox
Runt
Zinc finger (Sp1/KLF-like)
Prdx2
Prdx6
Casp3
Casp9
14
11
8
2
5
9
b
chr1:161,250,670-161,251,230
chr8:46,616,967-46,617,775
chr8:84,968,567-84,970,066
chr4:141,793,440-141,793,944
Ace-H3 C&T
WT+Saline 1
WT+Saline 2
WT+β-HB 1
WT+β-HB 2
KO+Saline 1
KO+Saline 2
KO+β-HB 1
KO+β-HB 2
30
0
Prdx6
Casp3
Prdx2
Casp9
d
TSS
Prdx2
chr8
+29 bp
Prdx6
chr1
+96 bp
Casp3
chr8
−11 bp
Casp9
chr5
−37 bp
Sp1/KLF motif
TSS
