# Supplementary material for: Postnatal ketone deficiency impairs adult oocyte quality via enhancing histone acetylation in malnourished mice
Source: J Biol Chem. 2026 Sep 8;302(10):113535. doi: 10.1016/j.jbc.2026.113535 (PMC13671086; doi:10.1016/j.jbc.2026.113535)
Supplement: Supplementary file 2 [file mmc2.pdf]

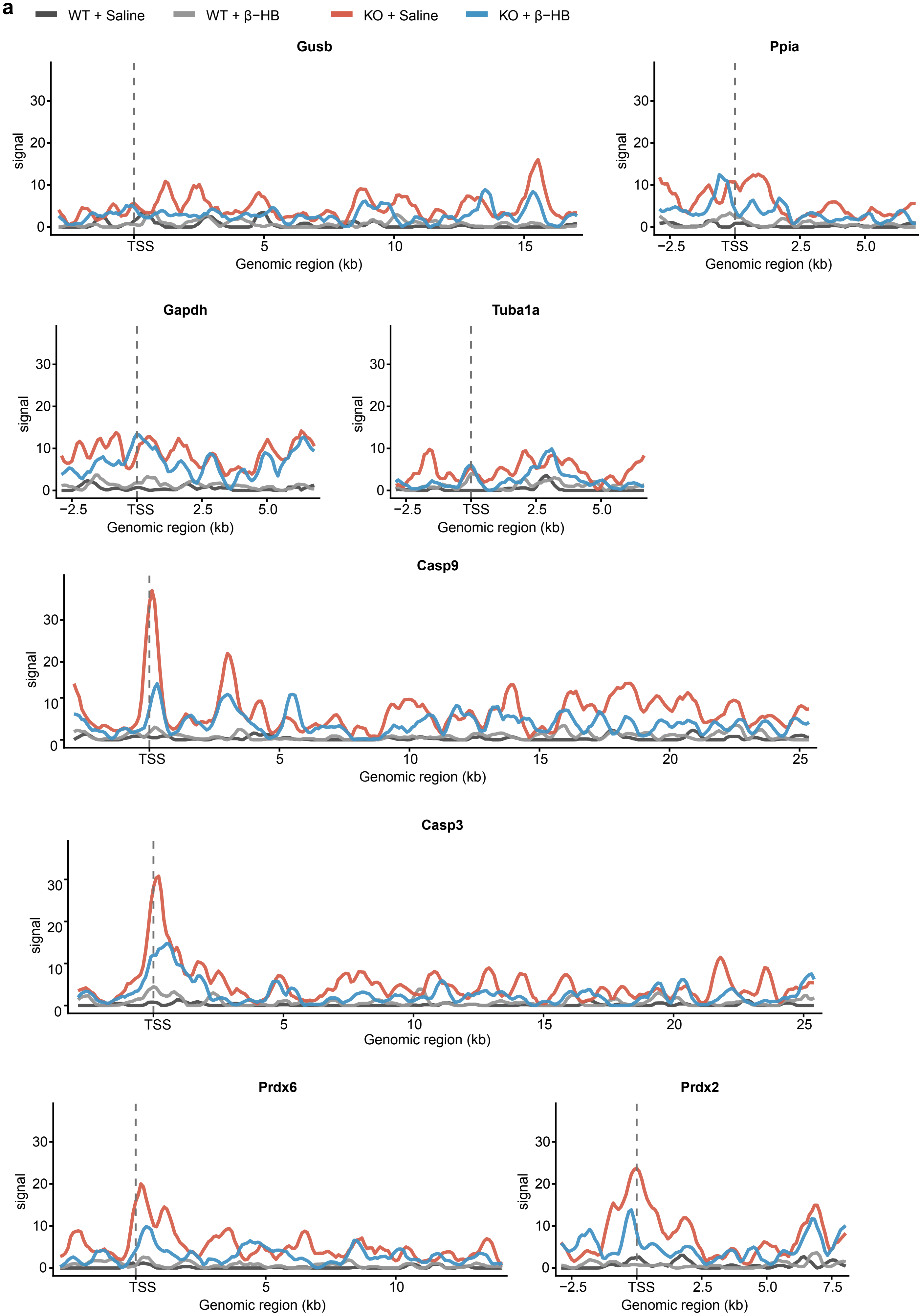
a
WT + Saline
WT + β−HB
KO + Saline
KO + β−HB
Gusb
Ppia
Gapdh
Tuba1a
Casp9
Casp3
Prdx6
Prdx2
signal
0
10
20
30
−2.5
TSS
2.5
5.0
7.5
5
10
15
20
25
Genomic region (kb)
