# Supplementary material for: Postnatal ketone deficiency impairs adult oocyte quality via enhancing histone acetylation in malnourished mice
Source: J Biol Chem. 2026 Sep 8;302(10):113535. doi: 10.1016/j.jbc.2026.113535 (PMC13671086; doi:10.1016/j.jbc.2026.113535)
Supplement: Supplementary file 3 [file mmc3.docx]

**Supplementary figure legends:**

**Fig. S1** **| Genomic annotation and motif features of differential Ace-H3-enriched regions associated with selected oxidative stress- and apoptosis-related genes.**

a, Genomic distribution of differential Ace-H3-enriched regions associated with Prdx2, Prdx6, Casp9, and Casp3. b, Representative Integrative Genomics Viewer (IGV) tracks showing normalized Ace-H3 CUT&Tag signals at selected oxidative stress- and apoptosis-related gene loci. Gray shading indicates promoter regions with increased Ace-H3 signals in KO oocytes. c, Motif features detected in promoter-associated differential Ace-H3-enriched regions linked to Prdx2, Prdx6, Casp3, and Casp9. Dot size represents motif count, and color intensity represents binding score. d, Schematic showing HOMER-detected Sp1/KLF-like motif positions within promoter-associated differential Ace-H3-enriched regions of Prdx2, Prdx6, Casp3, and Casp9. Red boxes indicate predicted Sp1/KLF-like motifs, and distances from the transcription start site (TSS) are labeled in base pairs. WT+Saline (n=2), KO+Saline (n=2), WT+β-HB (n=2), KO+β-HB (n=2).

**Fig. S2 | Gene-specific promoter-associated Ace-H3 enrichment patterns in KO oocytes.**

a, Per-gene Ace-H3 CUT&Tag signal profiles across the gene body with 3 kb upstream and downstream flanking regions for the four reference genes (Gusb, Ppia, Gapdh, Tuba1a) and the four target genes (Casp9, Casp3, Prdx6, Prdx2). WT+Saline, WT+β-HB, KO+Saline, and KO+β-HB oocytes are overlaid in each panel, and the signal was capped at 30. All data are presented as the mean ± S.D. WT+Saline (n=2), KO+Saline (n=2), WT+β-HB (n=2), KO+β-HB (n=2).
